# A systematic review of peer-reviewed literature authored by medical professionals regarding US biomedicine's role in responding to climate change

**DOI:** 10.1016/j.pmedr.2018.11.014

**Published:** 2018-11-24

**Authors:** Ross Graham, John Compton, Keith Meador

**Affiliations:** a Center for Biomedical Ethics and Society, Vanderbilt University Medical Center, Nashville, TN, United States of America; b Mental Health and Chaplaincy, Dept. of Veterans Affairs, Durham, NC, United States of America

**Keywords:** Climate change, Global warming, Ethics, Health policy, Environmental health, Epidemiology

## Abstract

Extant literature illustrates a substantive impact on human health because of climate change. Despite this, discussions of the ethical and policymaking role of US health care's response to this problem are underdeveloped within peer-reviewed literature indexed in core medical databases. We conducted a systematic literature review in August 2017 at Vanderbilt University Medical Center of the following medical, business and policy databases to examine the state of inquiry on this topic: PubMed, CINAHL, PsychINFO, JAMA Network, Health Affairs, Business Source Complete, Greylit.org, LexisNexis Academic, Proquest Dissertations and Theses Global. An initial sample of *n* = 4434 rendered *n* = 75 articles precisely addressing this question following a two-tiered systematic examination of content. US medical professionals were most concerned by the health impacts of air pollution and respiratory complications, extreme weather events, and rising infectious/vector-borne diseases. They were least concerned by rising rates of migration and stresses to sanitation systems. Medical professionals took a broadly proactive stance to the issue, highlighting the need to implement education and advocacy strategies. Politics was the least pertinent motivation for climate change-related recommendations. Furthermore, partnerships between health care and public agencies were identified as holding the greatest potential for meaningful change. Mitigation approaches were slightly more common than adaptation approaches. We conclude that, while the enthusiasm of the medical community is commendable, efforts to address climate change in US health care are overly fractured, and lack the necessary expertise for efficaciousness.

## Highlights

• Medical professionals showed overwhelming support for addressing climate change.
• Most frequently cited climate related health impacts were: air pollution, extreme weather, and infectious disease.
• Most common strategies involved education and/or advocacy.
• Literature favored partnerships between health care and the public sector to address climate change.

## 1 Introduction

The Lancet and University College, London (UCL) (Costello et al., 2008) report on climate change and health clearly demonstrates not only the urgent need for a multi-dimensional response to foster and preserve human health. It highlights the many direct health impacts of global climate change ranging from increased threats of extreme weather events, issues with food security and food safety, decreased air quality, to enhanced threats from vector-borne and infectious diseases and indirect consequences such as economic and population instability. Furthermore, tools to predict the consequences and pace of climate change are consistently rendered too conservative with every advent of new data (Hansen et al., 2016). It also argues that the pervasive nature of global climate change requires adaptation and mitigation responses that address energy use, carbon sequestration, and public health systems responses to climate related health impacts (Costello et al., 2008). As climate change is a human-caused reality (Oreskes, 2004) that threatens the foundations of human health and wellness (Patz et al., 2005), understanding the various ways in which diverse sectors engage this problem is a foundational exercise in designing an integrative approach (Enkvist and Rosander, 2007). This observation, in tandem with health care's authoritative position in American culture (Ludmerer, 2014) guides this systematic literature review. We ask how the American health care sector conceptualizes its role in addressing global climate change from policy discussions to clinical contexts. Moreover, we seek to characterize how this question is answered at the site where information is routinely acquired by working medical professionals – peer-reviewed literature indexed in major medical databases. The review highlights the required urgency of climate change efforts - these efforts should include both mitigation and adaptation strategies in the realms of patient and care provider education, advocacy in the public sector, and focused energy to reduce health care's carbon footprint while still meeting the increased care needs brought by climate change.

## 2 Method

This review is concerned with systematically investigating the role of US health care in responding to climate change from the vantage point of a health care professional routinely accessing medical literature. Accordingly, the data is limited to peer-reviewed literature indexed in medical databases – we do not claim a total encapsulation of responses by all health care entities. We seek a perspectival, rather than exhaustive, literature review.

To accomplish this, we first searched databases frequented by the following disciplines: physicians, nurses, administrators and legal counsel, environmental and public health professionals, non-M.D. medical academics, and students. These were PubMed, CINAHL, PsychINFO, JAMA Network, Health Affairs, Business Source Complete, Greylit.org, LexisNexis Academic, Proquest Dissertations and Theses Global. We employed MeSH terms that concurrently described three broad categories: global climate change, health/health care, and policy. A comprehensive breakdown of the search strategies is in the appendix. We further circumscribed the search by date, searching after Michael Mann and Raymond Bradley's 1999 ‘hockey stick graph’ publication (Mann and Hughes, 1999), which correlated an uptick in global temperatures with exponential increases in hydrocarbon combustion during the Industrial Revolution, and became a lightning rod for climate science deliberations in US politics. Articles had to be written by a US health care professional, specifically address climate change, advocate for a particular response from the US health care community, and be of article length. Pieces that discussed climate change only as a potential cause of a health-related phenomenon (e.g. an increase in Lyme's disease among a certain population) were excluded, since they primarily focused upon a specific pathology, rather than climate change as a multivariate issue. We also excluded unpublished or long-form works such as dissertations and books after the initial search had been conducted.

The search yielded 4426 articles that we uploaded into Covidence in order to complete the systematic review in accordance with PRISMA guidelines. Twenty-seven duplicate articles were immediately removed. Through Covidence, two authors reviewed the title and abstracts of each of the 4399 remaining articles. Disagreements were resolved through deliberative discussion. The inclusion criteria sent 401 articles to full-text review. This more detailed review process removed 334 more articles – 163 were concerned with non-domestic health care, 53 were not authored by health care professionals, 44 contained no explicit policy recommendation, 36 were primarily concerned with an environmental issue other than climate change, 23 further duplicates emerged, and 15 did not fit the literature format for inclusion. Sixty-seven articles were finally included for full analysis and categorization. Two authors independently read each article in their entirety and organized them by categories.

Categories were decided based on the following rationale. We recorded the professional specialty of each lead author, hypothesizing that the climate change policy purview of different health professionals would be influenced by their training and practice. For instance, we anticipated public health professionals would frame climate change in adaptive terms. We predicted that author location would prove influential, noting the extensive geographic variety in climate change beliefs and attitudes across the US (Howe et al., 2015). We categorized each article by broad motive for engagement with climate change, hypothesizing that health threats and economics would most likely incite action. We extracted any disease or health threat and placed it into eight health trend categories. This assessed what medical professionals expect to respond to in their practice as result of climate change, and whether this correlates with expected epidemiology. For example, we predicted that extreme weather events would be mentioned frequently, given their dramatic and visible character. Each article was examined for evidence of adaptation and/or mitigation approaches to the problem. We expected mitigation responses to be dominant, in light of widespread conceptualization that climate change is abstract and discountable in the immediate (Trope and Liberman, 2003). We recorded the sectors that an article proposed to intervene upon, anticipating a high frequency of suggested changes to energy sourcing and transportation. Following this, we ascertained *how* an article proposed to intervene, expecting the medical community to favor educational and technological strategies, as these are already great strengths of US medicine. Given the mosaic of institutions and entities that piece together US health care, we recorded any suggested collaborations and partnerships. These were broken up into the following categories - for-profit private entities, non-profit private entities, and public entities, predicting that public sector partnerships would be favored in accordance with US governments recently dominant voice on the issue. Finally, we evaluated the overall disposition of an article, observing how inactive, reactive or proactive their suggestions were, in order to get a high-level view of how much US health care is responsible for climate change.

**Exhibit 1:** Required definitions for categories.

Category	Definition
Approach	
Adaptation	Approach modeled around accepting and responding to definite current and/or future climate change impacts
Mitigation	Approach attempts to reduce rate and extent of as-yet-unrealized climate change by curbing carbon footprint

Motive	
Political	Driven to action by presence and/or absence of political will regarding climate change
Ethical	Driven to action by ethical imperative of climate change
Health trends	Driven to action by current and/or future health impacts of climate change
Economic	Driven to action by economic consequences of climate change

Pathology	
Infectious/vector-borne disease	Increased rate and morbidity of infectious, vector-borne, or other communicable disease caused by climate change
Food	Food system compromise, soil erosion/infertility, malnutrition or other food-related issue associated with climate change
Water	Drought, freshwater shortage, groundwater salination, aquifer depletion or other water-related issue associated with climate change
Housing/sanitation	Insufficient, destroyed or otherwise hazardous housing and sanitation caused by climate change
Extreme weather	Extreme heat, rainfall, hurricanes or other extreme weather event aligned with climate change
Migration	Domestic/international relocation, civil unrest or displacement resulting from climate change
Air pollution	Particulate matter and airborne toxins caused by industrial processes that induce climate change
Mental health	Anxiety, depression, addiction and other psychological ailments resulting from climate change impacts

Profession	
M.D.	Primary authorship by medical doctor
Nurses	Primary authorship by nurse
Non-M.D. medical academics	Primary authorship by medical academic e.g. anthropologist, ethicist
Environmental/public health	Primary authorship by environmental or public health practitioner
Administrators/lawyers	Primary authorship by administrators, lawyers or clerical
Students	Primary authorship by medical student

Role	
Leadership role	Health care should own, shape and promote best practice for addressing climate change, fully characterizing it as a health care concern
Proactive role	Health care should zealously implement best practice for addressing climate change, going above and beyond existing prescriptions and mandates
Conformative role	Health care should comply with existing prescriptions and mandates for addressing climate change
Passive/no role	Health care should take no action to address climate change

Region	
South	AL, AR, DE, FL, KY, LA, MD, MS, NC, OK, SC, TN, TX, VA, DC, WV
Northeast	CT, ME, MA, NH, NJ, NY, PA, RI, VT
Midwest	IL, IN, IA, KS, MI, MN, MO, NE, ND, OH, SD, WI
West	AK, AZ, CA, CO, HI, ID, MT, NV, NM, OR, UT, WA, WY

Sector	
Energy	Reduce carbon footprint via alternative energy sourcing/energy reduction
Food	Reduce carbon footprint of food consumed
Water	Reduce carbon footprint of water utilization
Waste	Reduce carbon footprint from waste generation/disposal
Infrastructure	Reduce carbon footprint from buildings and static infrastructure
Transport	Reduce carbon footprint from related transport

Partnerships	
None	Health care has no need to respond to climate change
Public	Health care should respond to climate change partnered with government/public entities
For-profit private	Health care should respond to climate change partnered with private, for-profit entities
Non-profit private	Health care should respond to climate change partnered with private, non-profit entities
Health care only	Health care should respond to climate change independently

Strategy	
Education	Education of professionals, public can/does help health care address climate change
Advocacy	Publicly highlighting risk, and supporting efforts/organizations reducing carbon can/does help health care address climate change
Technology	Technological solutions and innovation can/does help health care address climate change
Legislation	Lobbying for, and shaping, legislative initiatives can/does help health care address climate change
Behavior	Behavior change programs can/do help health care address climate change

The reviewer's category selections aligned 79% of the time, generating a Cohen's Kappa agreement coefficient of 0.53. Any differences were discussed and resolved, and results were tabulated. We selected seven articles that employed exhaustive reference lists (between fifty and two hundred citations) across a range of medical sub disciplines for hand searching (Maibach et al., 2008; Pinkerton et al., 2012; Hogrefe et al., 2004; Burke, 2016; Gould and Morello-Frosch, 2015; Sarfaty et al., 2014; Bedsworth, 2009). This process involved reviewing the citations of these articles and applying the same inclusion criteria and categorization process, yielding eight further articles. Seventy-five articles advanced to final categorization (see Exhibit 2).

Descriptive statistics were generated to illustrate broad trends. Chi-square tests analyzed predictive capacity of categorical variables – role, profession and region.

**Exhibit 2:**
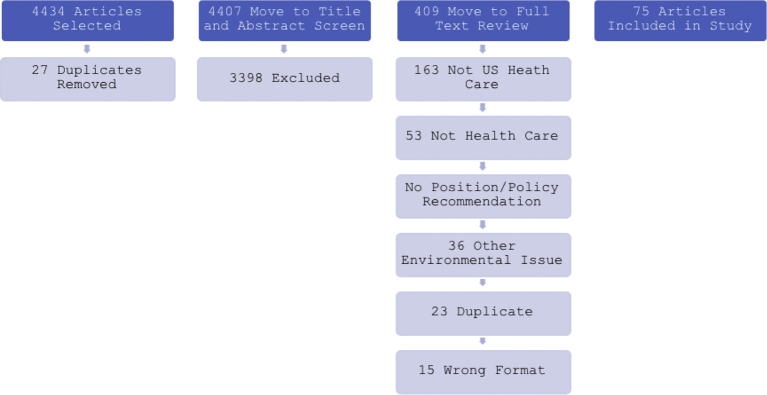
Flow chart of review process.

## 3 Results

We noted a substantial percentage of authors advocating for health care to take a proactive (59%) or leadership (32%) role in combatting climate change, as well as a broad engagement with both adaptation (76%) and mitigation (81%) strategies. The predominant health concerns motivating health care professionals were air pollution (83% articles), extreme weather events (77%) and infectious/vector-borne disease (75%). Less concern was evident over mental health (42%), migration (39%) and sanitation/housing (32%) issues caused by climate change. Education (92%) was overwhelmingly the preferred strategy for change. Advocacy measures were also regularly suggested (79%). With regards to carbon footprint reduction, authors focused upon energy sourcing (72%), most often in the form of renewable energy projects or schemes to reduce power utilization. Changes in food (36%), water (31%) and waste management (29%) systems were least common. Furthermore, 92% of the literature said climate-related health trends were motivation for engagement, whereas concerns about political issues were evident in only a third (33%) of the pieces. Health care professionals showed consistently strong support for partnerships with public institutions in order to achieve climate goals (81%). This compared with lesser support for partnerships with the private sector (24%), and even less for independent (i.e. no partnerships) strategies (12%).

**Exhibit 3:** Summary of overall results.

Cumulative variables		
Variable	No. of articles	% of articles
Total	75	100
Profession		
Doctors	16	21
Nurses	6	8
Environmental/public health	31	41
Administrators/lawyers	6	8
Non-M.D. medical academics	14	19
Students	2	3

Region		
South	31	41
Northeast	19	25
Midwest	10	13
West	15	20

Role		
Leadership role	24	32
Proactive role	44	59
Conformative role	6	8
No role	1	1


Non-cumulative variables		
Approach		
Adaptation	57	76
Mitigation	61	81

Pathology		
Infectious/vector-borne disease	56	75
Air pollution	62	83
Extreme weather	58	77
Housing/sanitation	24	32
Food	52	69
Water	50	67
Mental health	32	43
Migration	29	39

Motive		
Health trends	69	92
Political	25	33
Ethical	34	45
Economic	36	48

Intervention - sector		
Energy	54	72
Food	27	36
Water	23	31
Waste	22	29
Transport	38	51
Infrastructure	39	52

Intervention – strategy		
Education	69	92
Advocacy	59	79
Technology	45	60
Behavior	46	61
Legislating	45	60

Partnerships		
No health care	1	1
Health care alone	9	12
Health care and public sector	61	81
Health care and for-profit business	18	24
Health care and non-profit/NGO	32	43

## 4 Results by region

The plurality (41%) of included literature came from institutions based in the south, as defined by the US Census regions. We suspect this is primarily because the region includes both Atlanta and Washington D.C., administrative homes of many large public and non-profit health care organizations e.g. Center for Disease Control. Southerners mainly took a proactive stance towards tackling climate change (61%), as did authors from the Northeast (47%), Midwest (50%) and West (73%) – there was no significance in the relationship between role and region *X*^*2*^(9, n = 75) = 6.38, p > 0.05. All four regions identified education as the best intervention strategy, considered energy sourcing most opportune for a reduction in carbon footprint, and emphatically agreed that climate-induced health threats were the primary motivation for increased engagement. Among other motivating factors, ethical concerns were more common among Northeastern (52%) and Midwestern (60%) practitioners, whereas economic concerns prevailed for Southerners (73%) and those from the West (48%). All regions consistently highlighted three health concerns associated with climate change: (1) air pollution, which was the most common answer in the Northeast (84%) and West (100%), (2) infectious and vector-borne disease, which was the most common answer in Midwest (70%) and (3) extreme weather, which was the most common answer in the South (94%) and Midwest (also 70%). Partnership preference varied little by region.

**Exhibit 4:**
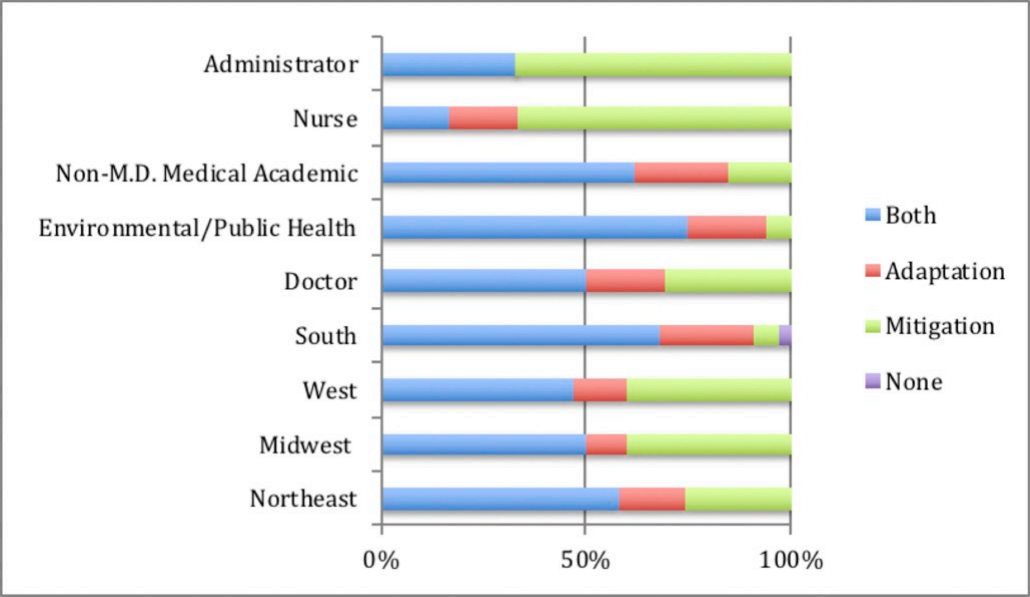
Chart displaying preferences for adaptation, mitigation, or combined climate change approaches among different medical professionals and different regional health institutions.

## 5 Results by role

Sixty percent of the total articles suggested health care should take a proactive role in fighting climate change. These proactive authors advocated for adaptation and mitigation strategies with equal regularity: 60% suggested a combination of both, while 20% recommended one or the other respectively. Authors primarily included environmental/public health practitioners (42%), followed by non-M.D. medical academics (22%) and physicians (20%), most commonly from a Southern institution (44%) – this relationship was not statistically significant, however *X*^*2*^(15, n = 75) = 23.63, p > 0.05. They commonly suggested that energy (71%), infrastructure (53%), and transport (47%) urgently required attention. They expressed concern regarding air pollution (84%), infectious and vector borne disease (80%), and extreme weather events (82%). Ninety-one percent cited health trends as a strong motivation for action, overshadowing economic (44%), ethical (40%) or political (36%) motivations. Many advocated for partnerships with government entities (84%) and/or NGO's (42%).

Twenty-four articles (32%) suggested health care should take a leadership role in addressing climate change. This literature advocated for adaptation strategies (8%), mitigation strategies (22%) or a combination of both (67%), and predominantly originated from Southern (38%) or Northeastern (33%) institutions. Notably, this group was dominated by practitioners of environmental/public health (88%), and supported education (96%) and advocacy (92%) efforts. Similar to the other groups, these practitioners commonly considered air pollution a pressing health concern (88%), while housing/sanitation (21%) concerns were less so. They also advocated for partnerships between health care and government entities more frequently (96%) than private or non-profit sectors.

Only one article suggested that health care had no role to play in ameliorating climate change. This author has significant ties to the Cato Institute, which has a history of climate change denial (Oreskes and Conway, 2010; Brulle, 2014) so we treat this data point with caution. We categorized six pieces (8%) as ‘conformative’ – they considered reducing the carbon footprint of energy and water systems a priority, and air pollution as the most pressing health concern available. All articles exhibiting a conformative position considered education to be the best mechanism available for change. Five of these authors were motivated by the health trends portended by climate change, while two thirds were motivated by potential economic and ethical ramifications.

## 6 Results by profession

The best-represented professional field was environmental and public health (n = 31, 41%). These practitioners were most likely to describe a proactive (61%) or leadership (35%) role, expressed concern about the health effects of air pollution (97%), extreme weather (94%), infectious disease (87%), and infirm arising from food and water issues (84% apiece). They most frequently advocated for education (97%) for combatting climate change, and were less likely to suggest legislative routes (42%). Energy (68%) and waste management (16%) were the largest and smallest opportunities for reduction of carbon footprint within health care, respectively. A majority (94%) was motivated to act by changing trends in health caused by climate change; economic concerns were the next most common motivating factor (52%). Due to the plethora of government public health agencies in either Washington DC or Atlanta, authors were often from the South (45%) and sought public sector alliances (87%) to address the problem.

**Exhibit 5:**
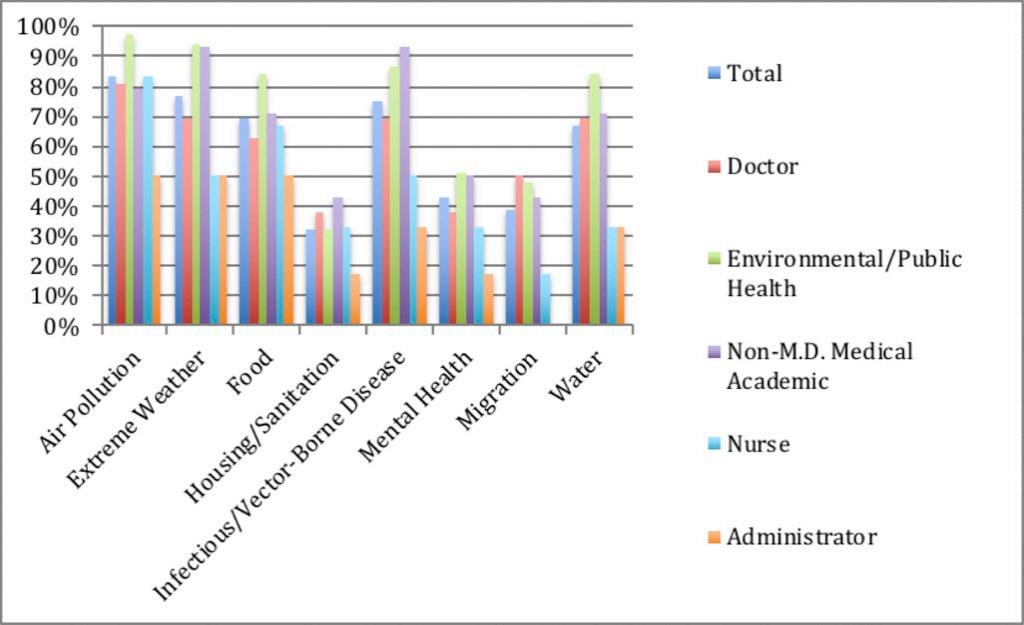
Chart displaying frequency of reference to different climate-related health impacts. Overall percentage, then divided by profession.

Of the sixteen articles written by physicians, fifteen advocated for a proactive (Trope and Liberman, 2003) or leadership (Ludmerer, 2014) role for medicine in fighting climate change. This group showed urgency for improving energy practices (75%), expressed concern about health conditions caused by increasing air pollution (81%), and commonly cited education as an antidote climate change (88%). These authors widely called for partnerships between health care and government agencies (81%). All considered changing health trends (100%), and over half considered ethical commitments (56%) as prerogatives for action.

**Exhibit 6:**
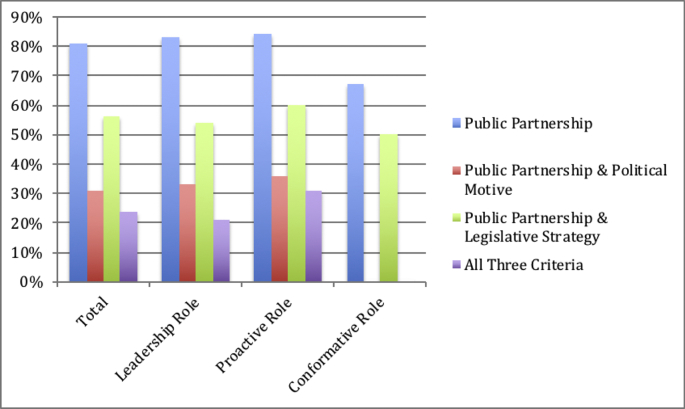
Chart displaying combinations of public strategies (public partnership, political motive, legislative strategy) for addressing climate change, divided by role.

Fourteen authors (19%) were categorized as non-M.D. medical academics; these largely called for proactivity (64%) or leadership (21%) from health care, with a preference for public partnerships (79%). They talked of the urgency of energy reform (64%), the effectiveness of education (93%) and the motivating quality of observable climate-related health trends (86%). Notably, this was the only group that identified extreme weather and infectious disease (93% apiece) as the most pressing health catalyst, which accords with prior epidemiological literature (Costello et al., 2008; Patz et al., 2005). The other groups of authors were nurses (n = 6), students (n = 2), and administrators/counsel (n = 6). Despite small sample sizes, it was notable that both nurses and administrators were strongly inclined to favor mitigation-based strategies over adaptation-based ones. Otherwise, these groups largely mirrored the wider dataset.

## 7 Discussion

US health care is a mosaic of elements. Government administers various programs and regulates, while a variety of for-profit businesses, non-profits and NGO's create their own health care infrastructures or augment the publicly administered ones. US health care is therefore necessarily collaborative. As addressing climate change requires extensive, uniquely complex forms of collaboration, the views of health care professionals on these collaborations are critical. There is substantial emphasis put upon the peer-reviewed medical literature in shaping views and policy. We observe an overwhelming preference for the public sector to provide primary support to health care for climate change activities. Furthermore, we expected this predilection to yield more emphasis on both political motivation and legislative strategy. Though this group was more likely to describe political motivation (33%) and advocate legislative strategy (69%) than authors who did not suggest partnering with the public sector (14%, 21%, respectively), they nevertheless preferred other strategies like education (95%) and advocacy (80%). This pattern remained consistent across stated roles with the exception of authors in the conformative role category, who never expressed political motivations (see Exhibit 6). Consequently, we have concerns about the cohesion between ideals and proposed strategy. The data suggests here that medical professionals generalize climate change as a public sector problem, but that the pathways by which the public sector creates social and policy changes is subsequently under-regarded. Incomplete sentiments like these are risky. The cultural authority of US health care is such that the strategies it espouses have reverberating effects across contexts. This reality calls for the medical community to have a thorough and integrative understanding of how to approach climate change.

With the exception of non-M.D. medical academics, air pollution was the most frequently mentioned health concern across all professional categories (see Exhibit 5). Though air pollution is a serious concern, predicted impact on health and morbidity is small when compared to that of infectious disease, extreme weather, or food security in the US. The US’ temperate climate and larger urban population exacerbate the risk of these phenomena (Costello et al., 2008; Patz et al., 2005). Additionally, collapses in grain production as a result of rising temperatures will immensely affect health outcomes (Burke, 2016) In fact, deaths related to respiratory disease are perhaps more likely to occur due to rising ambient air temperature than through particulate matter from air pollution (Patz et al., 2005), especially if urbanization continues apace. Therefore, we suggest that air pollution receives inflated concern in light of other, more complex and epidemiologically significant consequences of climate change. We are concerned that this again betrays significant misunderstanding of the threats climate change pose to US health care and society more generally. Four authors within this review shared this concern, outlining worrisome lack of knowledge, information, or expertise regarding climate change in the medical community (Gould and Morello-Frosch, 2015; Sarfaty et al., 2014; Bedsworth, 2009; Maibach et al., 2008). Because American culture views the medical community as trusted professionals, these systemic misunderstandings threaten to negatively impact awareness and action. We believe that further research is necessary to substantiate this idea and recommend concrete proposals, such as systemic modifications, patient education and climate-focused CME training.

The strategies authors most readily supported tended to be future-oriented, with advocacy (78%) and education (92%) strategies most popular among the articles. Public advocacy certainly positions a health care entity to support efforts that address climate change, but ultimately outsources the need to act. We realize that it is beyond the scope of organizations to care about all issues at all times, but health care has an unimpeachable empirical, ethical and fiscal stake in climate change. There was a strong emphasis on education, too, reflecting the demonstrable value in developing research proposals to gather more data (Pinkerton et al., 2012), curricula to teach and form future care providers (Friedrich, 2017), or educating patients (Sarfaty et al., 2014). The immediacy of climate change requires a strong response to current practice. Care providers right now need to be equipped with the tools to address health impacts of climate change when it presents in the clinic (Bedsworth, 2009). Therefore, care providers need not only education around the basic science of climate change, but the clinical phase of their education needs to appropriately synergize prevailing trends in health (e.g. outbreaks in Lyme's disease) directly to the broader phenomenon when appropriate.

Because of the advanced, interconnected nature of global climate change, an integrative response must include both adaptation and mitigation strategies (Hawken, 2017; Lazarus, 2008). We found that articles from administrators and nurses deviated from this notion by only calling for mitigation efforts (both at 67%). This could be because mitigation strategies consider climate change to be essentially unrealized and thus ultimately solvable. Framing climate change this way has great currency, because it is more hopeful and thus can effectively mobilize action (McKenzie-Mohr, 2011). Nevertheless, a mitigation-only response ignores the often neglected functional need to adapt to the effects of climate change that are already here (Pielke Jr et al., 2007). For example, one article from a health care administrator (Jarousse, 2012) discusses ways hospitals can implement environmentally friendly waste disposal practices, highlighting that health care generates over 6500 tons of waste per day nationally. This article details the cost-saving and brand-development benefits of recycling, composting, and source reduction, specifically mentioning that sustainability enhances public perception. The author does highlight that waste programs can lay the foundation for more challenging sustainability efforts, but the examples she lists are further mitigation practices such as energy reduction, food sourcing, and water use. The article profiles three hospitals that have made significant strides in waste reduction, and each hospital gives primacy to the cost savings rather than emission reductions or other ecological measures. Financial sustainability is a vital component to health care management, but the mitigation strategies listed in this piece seem to serve the ends of brand development rather than environmental sustainability. This economic emphasis may be strategic to appeal to wider audiences, but it dangerously advocates for a mitigation-only response. Focusing only on mitigation strategies ignores the unfortunate reality that a nuanced approach to global climate change must include both emission reductions and adaptive strategies that ensure human health in the face of inevitable climate change. Furthermore, the paradigmatic shift necessary for combatting climate change has been achieved before in US health care, notably through germ theory and smoking cessation campaigns. We believe that limited attempts at financial incentive are conceptually insufficient to catalyze similarly radical changes in the future.

## 8 Conclusion

Medical professionals have a substantial role to play in addressing the consequences of climate change. The nature of this role will be decided, in part, by how active or passive the approach of the medical community is. The divergence apparent in the peer-reviewed medical literature between the coming health consequences of climate change and policy decisions that are insufficient to address it will be catastrophic if realized. This literature review establishes broad themes for health care professional browsing their native literature on the relationship between climate change and US health care that beg further research. High levels were evident of enthusiasm and drive among medical professionals to act as agents for change on an individual and collective level, favoring the tools of education and advocacy to counter the urgency of various well-articulated public health concerns. These professionals were most alarmed by how rising air pollution, extreme weather, and increasingly ideal conditions for infectious/vector-borne diseases will damage US public health. Furthermore, they largely felt health care should forge partnerships with public and/or non-profit entities to tackle the problem, with greatest effort being dedicated towards revitalizing health care's energy resources. We call for further research, however, on the gap evident between high enthusiasm and inconsistent or ineffective strategies. For example, a factor discussed sparingly in this review, but with undoubtedly enormous consequences, is the threat posed by potential food system collapse (Patz et al., 2005). For another, an emphasis on education and advocacy suggests that the perceived temporal urgency climate change poses to health care is still somewhat underestimated. Furthermore, the literature exhibited enthusiasm for partnering with public institutions, presumably to engage capable stakeholders in advocating for legislative change or addressing the political gridlock climate change embodies at multiple levels of governance. Further research on these themes can help the medical community orient their practices towards effectively addressing the problem. The many articles circulating on the topic of climate change and health gives us hope that health care will live into its powerful tradition of appropriately responding to human health concerns.

Kilmer and MacCoun (2017), in a recent review, outline how critical the medical community has been in advocating, normalizing and explaining the benefits of medical marijuana to US populations. Many positive efforts have resulted in criminal justice, local economic, educational, and public receptivity to evidence-based social policy challenges. We believe this illustrates the substantive role health care still plays in shaping broader social, political and ethical trends. We hope that an analogue can be drawn for the approach health care takes towards climate change, and stress the need for similar levels of acuity and resolve to be further emphasized in their professional literature.

## Conflicts of interest

None.

## Funding

This research did not receive any specific grant from funding agencies in the public, commercial, or not-for-profit sectors.

## Appendix A Supplementary data

Articles included in review Image 1

Search strategy Image 2

Coding and raw data Image 3
