# Supplementary material for: A systematic review of peer-reviewed literature authored by medical professionals regarding US biomedicine's role in responding to climate change
Source: Prev Med Rep. 2018 Nov 24;13:132–8. doi: 10.1016/j.pmedr.2018.11.014 (PMC6299145; doi:10.1016/j.pmedr.2018.11.014)
Supplement: Supplementary file 1 — Articles included in review [file mmc1.docx]

1. Afzal BM. Global warming: a public health concern. Online Journal of Issues in Nursing. 2007;12(2):6p-p.
2. Allen M, Akpinar-Elci M. Changing Discourses in climate health: An anti-disciplinary perspective. Ecohealth. 2016;13(1):4-5.
3. Anstey MH. Climate change and health - what's the problem? Globalization & Health. 2013;9:4.
4. ASTHO. State and territorial health agency needs for a changing climate. ASTHO Report. 2012;1:1-14.
5. Auerbach PS. Physicians and the Environment. Jama. 2017;299(8):956-8.
6. Balbus J, Ebi K, Finzer L, Malina C, Chadwick A, McBride D, Chuk M, Maibach E. Are we ready? Preparing for the public health challenges of climate change. 2008. Environmental Health Fund Report;1:1-31.
7. Balbus J. What does climate change have to do with human health? with John Balbus. Environ Health Perspect. 2011;119(9):1 preceding a382.
8. Bauchner H, Fontanarosa PB. Climate Change: A Continuing Threat to the Health of the World’s Population. Jama. 2017;312(15):1519.
9. Bedsworth L. Preparing for climate change: A perspective from local public health officers in California. Environ Health Perspect. 2009;117(4):617-623.
10. Benjamin GC. 2017 #NPHW and Beyond: Climate Changes Health. Am J Public Health. 2017;107(5):630.
11. Bernard SM, Ebi KL. Comments on the process and product of the health impacts assessment component of the national assessment of the potential consequences of climate variability and change for the United States. Environ Health Perspect. 2001;109 Suppl 2:177-84.
12. Bouley T. Health benefits of policies to mitigate climate change...Lancet. 2010 Nov 27;376(9755):1801-2; Lancet. 2010 Nov 27;376(9755):1802-4. Lancet. 2011;337 North American Edition(9770):996-8.
13. Brown L. Preparing for the Public Health Challenges of Climate Change: Perspectives From Local Public Health. Journal of public health management and practice : JPHMP. 2016;22(1):102-4.
14. Bunyavich S, Landrigan CP, McMichael AJ, Epstein PR. The impact of climate change on child health. Ambulatory Pediatrics. 2003;3(1):44-52
15. Chalupka S. Climate Change and Health...Laura Anderko, PhD, RN. American Journal of Nursing. 2014;114(8):67-9.
16. Chenven L, Copeland D. Front-line worker engagement: greening health care, improving worker and patient health, and building better jobs. New solutions : a journal of environmental and occupational health policy : NS. 2013;23(2):327-45.
17. Cohen G. How Health Care Can Lead The Way on Renewable Energy. Health progress (Saint Louis, Mo). 2016;97(3):20-4.
18. Cooney CM. Preparing a People: Climate Change and Public Health. Environ Health Perspect. 2011;119(4):A166-71.
19. Crowley RA. Climate Change and Health: A Position Paper of the American College of Physicians. Ann Intern Med. 2016;164(9):608-10.
20. DeNicola E, Subramaniam PR. Environmental attitudes and political partisanship. Public Health. 2014;128(5):404-9.
21. Diaz JH. The Public Health Impact of Global Climate Change. Family & Community Health: The Journal of Health Promotion & Maintenance. 2004;27(3):218-29.
22. Ebi K, Balbus J, Kinney P, Lipp E, Mills D, O’Niel M, Wilson M. U.S. funding is insufficient to address the human health impacts of and public health repsonses to climate variability and change. Environmental Health Perspectives. 2009;117(6):857-862.
23. Ebi K. Climate Change And Health Risks: Assessing And Responding To Them Through ‘Adaptive Management’. Health Affairs. 2011;30(5):924-30.
24. Ebi KL, Hess JJ, Isaksen TB. Using Uncertain Climate and Development Information in Health Adaptation Planning. Current environmental health reports. 2016;3(1):99-105.
25. Eidson M, Clancy KA, Birkhead GS. Public Health Climate Change Adaptation Planning Using Stakeholder Feedback. Journal of public health management and practice : JPHMP. 2016;22(1):E11-9.
26. Friedrich MJ. Medical Community Gathers Steam to Tackle Climate’s Health Effects. Jama. 2017;317(15):1511-3.
27. Frumkin H, Hess J, Luber G, Malilay J, McGeehin M. Climate change: the public health response. Am J Public Health. 2008;98(3):435-45.
28. Goklany IM. Global health threats: global warming in perspective. Journal of American Physicians & Surgeons. 2009;14(3):69-75.
29. Gomez A, Balsari S, Nusbaum J, Heerboth A, Lemery J. Perspective: Environment, biodiversity, and the education of the physician of the future. Academic medicine : journal of the Association of American Medical Colleges. 2013;88(2):168-72.
30. Gould S, Rudolph L. Challenges and Opportunities for Advancing Work on Climate Change and Public Health. International journal of environmental research and public health. 2015;12(12):15649-72.
31. Hall AG. Promoting environmental health: advocating for patient safety. Nurs Adm Q. 2010;34(4):278-9.
32. Hampton T. Hospitals and clinics go greenb for health of patients and enviornment. Medical News & Perspectives. 2007;298(14):1625-29.
33. Harris N, Pisa L, Talioaga S, Vezeau T. Hospitals going green: a holistic view of the issue and the critical role of the nurse leader. Holistic Nursing Practice. 2009;23(2):101-11.
34. Hess JJ, Heilpern KL, Davis TE, Frumkin H. Climate change and emergency medicine: impacts and opportunities. Academic Emergency Medicine. 2009;16(8):782-94.
35. Hess JJ, McDowell JZ, Luber G. Integrating Climate Change Adaptation into Public Health Practice: Using Adaptive Management to Increase Adaptive Capacity and Build Resilience. Environ Health Perspect. 2012;120(2):171-9.
36. Jackson R, Shields KN. Preparing the U.S. health community for climate change. 2008;29:57-73.
37. Jarousse LA. Environmental Sustainability Programs for HOSPITALS. H&HN: Hospitals & Health Networks. 2012;86(1):33-41.
38. Kansas Nursing Association. 2009 resolutions: Global climate change and human health. Kansas Nurse. 2008;83(9):10.
39. Keim ME. Building human resilience: The role of public health preparedness and repsonse as an adaptation to climate change. 2008;35(5):508-516.
40. Keim ME. Preventing disasters: public health vulnerability reduction as a sustainable adaptation to climate change. Disaster medicine and public health preparedness. 2011;5(2):140-8.
41. Koh H, the former Massachusetts commissioner of public h, the 14th assistant secretary for health for the USDoH, Human Services. A quadruple-boarded physician DKhpmtaim, public health literature emtafiaiph, has received 5 honorary doctorate degrees HVFPotPoPHLatHTHCSoPH, et al. Communicating the Health Effects of Climate Change. Jama. 2017;315(3):239-40.
42. Krueger J, Biedrzycki P, Hoverter SP. Human health impacts of climate change: implications for the practice and law of public health. The Journal of law, medicine & ethics : a journal of the American Society of Law, Medicine & Ethics. 2015;43 Suppl 1:79-82.
43. Maibach EW, Chadwick A, McBride D, Chuk M, Ebi KL, Balbus J. Climate change and local public health in the United States: preparedness, programs and perceptions of local public health department directors. PLoS One. 2008;3(7):e2838.
44. Maibach EW, Roser-Renouf C, Leiserowitz A. Communication and marketing as climate change-intervention assets: A public health perspective. Am J Prev Med. 2008;35(5):488-500.
45. McCarthy M. Obama warns of climate change threat to public health. BMJ: British Medical Journal (Clinical Research Edition). 2015;350:h1947-h.
46. McCartney PR. Climate change and child health. MCN The American journal of maternal child nursing. 2007;32(4):255.
47. McGinty M. A Conversation with Dr. John Holdren, Assistant to the President for Science and Technology, Director of the White House Office of Science and Technology Policy. Health security. 2016;14(2):40-2.
48. National Environmental Health Association. Policy statement: Climate change. 2017. Available at http://neha.org/sites/default/files/eh-topics/climate-change/NEHA_Policy_Statement_Climate_Change_BOD_Adopted_Final.pdf
49. Patz J, Campbell-Lendrum D, Gibbs H, Woodruff R. Health impact assessment of global climate change: expanding on comparative risk assessment approaches for policy making. 2008;29:27-39.
50. Patz JA, Frumkin H, Holloway T, Vimont DJ, Haines A. Climate change: challenges and opportunities for global health. JAMA: Journal of the American Medical Association. 2014;312(15):1565-80.
51. Perera FP. Multiple Threats to Child Health from Fossil Fuel Combustion: Impacts of Air Pollution and Climate Change. Environ Health Perspect. 2017;125(2):141-8.
52. Pinkerton KE, Rom WN, Akpinar-Elci M, Balmes JR, Bayram H, Brandli O, et al. An official American Thoracic Society workshop report: Climate change and human health. Proc Am Thorac Soc. 2012;9(1):3-8.
53. Plough AL. Global News. Am J Public Health. 2016;106(11):1896.
54. Radtke T, Gist GL, Wittkopf TE. National Environmental Health Association position on global climate change. J Environ Health. 2001;64(2):30-2.
55. Resnik DB. Bioethics and Climate Change: A Response to Macpherson and Valles. Bioethics. 2016;30(8):649-52.
56. Ring W. Climate 911: A Call to Action for Health Professionals. International Journal for Human Caring. 2014;18(2):43-4.
57. Robert Wood Johnson Foundation. Making the connection : linking policies to prevent climate change and childhood obesity. 2011. Robert Wood Johnson Repoert;1:1-8.
58. Sarfaty M, Abouzaid S. The physician's response to climate change. Family medicine. 2009;41(5):358-63.
59. Sarfaty M, Mitchell M, Bloodhart B, Maibach EW. A survey of African American physicians on the health effects of climate change. International journal of environmental research and public health. 2014;11(12):12473-85.
60. Schwartz BS, Parker C, Glass TA, Hu H. Global environmental change: what can health care providers and the environmental health community do about it now? Environ Health Perspect. 2006;114(12):1807-12.
61. Serb C. Think green. Hospitals & health networks. 2008;82(8):22-6, 35, 1.
62. Shea KM. Climate change: public health crisis or opportunity. Journal of public health management and practice : JPHMP. 2008;14(5):415-7.
63. Shea KM. Global climate change and children's health. Pediatrics. 2007;120(5):1149-52.
64. Sheffield PE, Landrigan PJ. Global climate change andae children's health: threats and strategies for prevention. Environ Health Perspect. 2011;119(3):291-8.
65. Smith JA, Vargo J, Hoverter SP. Climate Change and Public Health Policy. Journal of Law, Medicine & Ethics. 2017;45:82-5.
66. Staropoli JF. The public health implications of global warming. Journal of the American Medical Association. 2002;287(17):2282.
67. Trust for America’s Health. Climate change: The public health response. 2014. Available at: http://healthyamericans.org/health-issues/wp-content/uploads/2014/08/Climate-Change-fact-sheet.pdf.
68. Valles SA. Bioethics and the framing of climate change’s health risks. Bioethics. 2013;27:305-308.
69. Vermont Nurse Connection. Health Care Without Harm announces sustainable food in health care awards: Awards recognize hospitals and staff implementing sustainable food programs. Vermont Nurse Connection. 2012;15(1):4.
70. Voelker R. Climate Change Puts Children in Jeopardy. Jama. 2017;301(21):2197-9.
71. Watson RT, Patz J, Gubler DJ, Parson EA, Vincent JH. Environmental health implications of global climate change. Journal of environmental monitoring : JEM. 2005;7(9):834-43.
72. Wellbery C, Department of Family Medicine GUMC, American Family Physician WDC. Code Green. Jama. 2017;312(12):1201-2.
73. Yellowlees P. Green healthcare: what does the future hold? Medscape journal of medicine. 2008;10(7):163.
74. Yellowlees P. Green healthcare: what is happening now? Medscape journal of medicine. 2008;10(7):162.
75. Yellowlees PM, Chorba K, Burke Parish M, Wynn-Jones H, Nafiz N. Telemedicine can make healthcare greener. Telemedicine journal and e-health : the official journal of the American Telemedicine Association. 2010;16(2):229-32.
